# The effect of civil and military flights on coagulation, fibrinolysis and blood flow: insight from a rat model

**DOI:** 10.1186/s12959-020-00237-8

**Published:** 2020-10-06

**Authors:** Anna Levkovsky, Rima Dardik, Daniel Barazany, David M. Steinberg, Mark Dan Kirichenko, Sara Apter, Edna Peleg, Daniel Silverberg, Ehud Grossman, Ophira Salomon

**Affiliations:** 1 Thrombosis Unit Sheba Medical Center, Coagulation Institute, 52621 Tel Hashomer, Israel; 2 Sackler Faculty of Medicine, Tel Aviv University, Tel Aviv, Israel; 3 National Hemophilia Center and Thrombosis Unit, Sheba Medical Center, Tel-Hashomer, Israel; 4 Strauss Computational Neuroimaging Center, Tel Aviv University, Tel Aviv, Israel; 5 Department of Statistics and Operations Research, Faculty of Exact Sciences, Tel Aviv University, Tel Aviv, Israel; 6 Department of Diagnostic Imaging, Sheba Medical Center, Tel-Hashomer, Israel; 7 Tel Aviv University, Tel Aviv, Israel; 8 Hypertension Unit, Sheba Medical Center, Tel-Hashomer, Israel; 9 Department of Vascular Surgery, Sheba Medical Center, Tel-Hashomer, Israel; 10 Internal Medicine Department, Sheba Medical Center, Tel Hashomer, Israel

**Keywords:** D-dimer, Hypobaric conditions, IL-6, MRI, Thrombin–antithrombin

## Abstract

**Background:**

Air travel thrombosis continues to be a controversial topic. Exposure to hypoxia and hypobaric conditions during air travel is assumed a risk factor. The aim of this study is to explore changes in parameters of coagulation, fibrinolysis and blood flow in a rat model of exposure to hypobaric conditions that imitate commercial and combat flights.

**Methods:**

Sixty Sprague-Dawley male rats, aged 10 weeks, were divided into 5 groups according to the type and duration of exposure to hypobaric conditions. The exposure conditions were 609 m and 7620 m for 2 and 12 h duration. Blood count, thrombin– antithrombin complex, D-dimer, interleukin-1 and interleukin-6 were analyzed. All rats went through flight angiography MRI at day 13-post exposure.

**Results:**

No effect of the various exposure conditions was observed on coagulation, fibrinolytic system, IL-1 or IL-6. MRI angiography showed blood flow reduction in lower limb to less than 30% in 50% of the rats. The reduction in blood flow was more pronounced in the left vessel than in the right vessel (*p* = 0.006, Wilcoxon signed rank test). The extent of occlusion differed across exposure groups in the right, but not the left vessel (*p* = 0.002, *p* = 0.150, respectively, Kruskal-Wallis test). However, these differences did not correlate with the exposure conditions.

**Conclusion:**

In the present rat model, no clear correlation between various hypobaric conditions and activation of coagulation was observed. The reduction in blood flow in the lower limb also occurred in the control group and was not related to the type of exposure.

## Introduction

Air travel thrombosis is a subgroup of thrombosis that occurs within 4 weeks following long haul air travel [1]. Exposure to hypoxia and hypobaric conditions during air travel are considered as risk factors, alongside immobilization, which is common to all land travel thrombosis cases [1–4].

Commercial airplanes usually fly at about 10,800 m (35,433 ft) above sea level, while compressing air to about 75.8 kPa (570 mmHg), which is essential to prevent hypoxia because of reduced barometric pressure at such an altitude. The cabin’s pressure is kept equivalent to an altitude of 1500-2500 m with partial oxygen pressure of 16.7 kPa (125 mmHg). Oxygen saturation is reduced to 90–93% in healthy individuals, but it may drop even to 80% during the flight in patients with pulmonary and/or heart disease.

Combat aircraft may operate at altitudes of 7620 m (25,000 ft) and even more [5], and the grade of air pressurization is dependent on the altitude [3, 5, 6].

The phenomenon of air travel thrombosis as a concept continues to be a controversial topic since most studies were conducted on few participants with heterogeneous clinical characteristics, type of exposure and duration, with no pre- and post-exposure comparison of coagulation parameters and lack of a control group [3, 7]. Furthermore, there was inconsistency concerning the activation of coagulation and fibrinolytic pathways when fragment 1 + 2, thrombin-antithrombin (TAT) complex and D-dimers were measured [3, 7–10].

The incidence of deep vein thrombosis (DVT) in low risk travelers was 1.6% compared to 5% in those with additional risk factors [11, 12].

Concerning hypobaric conditions, there was a transient activation of coagulation factors in volunteers held in a hypobaric chamber [13]. In rabbits, the risk of DVT was augmented by exposure to hypobaric conditions following surgery of the femur, as compared to rabbits that were not exposed to a postoperative drop in air pressure [14].

Hypoxia has been demonstrated to decrease fibrinolytic activity and incite the formation of oxygen free radicals and nitric oxide by endothelial cells [7]. The latter causes relaxation of the venous vessels with decreased blood flow velocity and stasis, which may promote venous thromboembolism.

Direct evidence that would support or exclude the association between air travel and thrombosis would require a large number of participants due to the low incidence of air-travel thrombosis, and it is unlikely that airline companies or funding agencies would sponsor such studies.

Therefore, in this study we used a rat model of exposure to hypobaric conditions compatible with conditions present during commercial and combat flights to explore changes that may occur in the coagulation, fibrinolysis and blood flow.

## Materials and methods

### Animals

Sprague-Dawley male rats were purchased from Envigo RMS (Jerusalem, Israel) at the age of 10 weeks and weight 340–405 g. The rats were acclimated for 3 days before study initiation. Three rats per plastic cage were housed at a temperature of 22 ± 2 °C in a controlled room with alternating 12-h light/dark cycle, with regular chow diet and water available ad libitum before and after the exposure experiment. During exposure to hypobaric conditions, smaller cages, housing two rats each, were needed to adjust to the cabin settings. The rats received regular diet but no water as the water bottles were not adjusted to the hypobaric conditions. The control rats were also deprived of water for 12 h, which corresponded to their time of exposure. All rat procedures were carried out based on ethical approval and in accordance with The Animal Care and Use Committee of Sheba Medical Center, Tel-Hashomer (approval number 1046/16).

### Study design

Groups of 12 rats each were assigned to the five groups defined by the type and duration of exposure to hypobaric conditions as shown in Table 1.

**Table 1:** Characteristic of rats’ group according to type and duration of exposure to hypobaric conditions

Groups	Height	Duration of exposure
1	609 m (2,000 ft)	2 h
2	609 m (2,000 ft)	12 h
3	7620 m (25,000 ft)	2 h
4	7620 m(25,000 ft)	12 h
5	Sea levels	12 h

### Hypobaric exposure

Rats were exposed to hypobaric conditions at the Israel Air Force Aeromedical Center’s Hypobaric Training facility. The chamber, purchased from Vacudyne (Chicago, USA), was connected to a vacuum pump with controlled air inflow and outflow. The hypobaric conditions were launched by dropping the barometric pressure. The low altitude condition in the study was equivalent to 609 m and represents atmospheric pressure of 706 mmHg, and 148 mmHg oxygen partial pressure (effective oxygen 19.4%, by measurement). The high altitude condition was equivalent to 7620 m and represents atmospheric pressure of 282 mmHg and 59 mmHg oxygen partial pressure (effective oxygen 7.6%, by measurement).

### Blood samples

Three mL blood was drawn from the retro-orbital sinus of each rat and divided into 3 tubes with 10% ethylenediaminetetraacetic acid (EDTA) following anesthesia (with 0.5 ml isoflurane), at day 4 before exposure to hypobaric conditions, at day 6 immediately post exposure and at day 21 a day before MRI was done. Blood count was performed using Beckman Coulter DxH900 analyzer (Farminpex N. V, USA).

#### Thrombin-Antithrombin (TAT) complex and D-dimer tests

Blood samples were centrifuged at 1000 x g for 15 min at room temperature, followed by additional centrifugation of the plasma at 10,000 x g for 15 min at 4 °C. Blood samples were processed within 1 h of blood withdrawal and stored at − 80 °C until analysis at the end of the study. Rat enzyme-linked immunosorbent assay (ELISA) kit (Mybiosource company, San Diego, USA) and rat D-dimer (D2D) competitive ELISA kit (Mybiosource company) were used for analyzing TAT complex and D-dimer, respectively, according to the manufacturer’s instructions.

#### Cytokines

Interleukin-1 (IL-1) and interleukin- 6 (IL-6) were analyzed by rat interleukin-1β and rat IL-6 ELISA kit (Mybiosource company) according to the manufacturer’s instructions.

### MRA screening

All rats went through MRI angiography (MRA) scan at Strauss MRI Center, Tel Aviv University, 13 days after hypobaric exposure using a 7 T/30 Bruker Biospec scanner with a 72 mm cylinder transmit-receive coil. The rats were placed in prone position in the scanner with the back limbs secured by tape. Anesthesia was maintained with isoflurane (2–3% in pure O_2_) and body temperature was kept at 38.5 °C by a heating pad during imaging. We used 2-dimensional Time of Flight (2D-TOF) angiography through TR/TE = 20 ms/2.25 ms, flip angle was 80°, 39 coronal slices of 1 mm with an in-plane resolution of 0.275 mm2 in a scan time of 7 min and 30 s. The 2D-TOF MRA images were acquired perpendicular to the orientation of the vessels’ blood flow.

### MRA score

TOF MRA produces an enhanced signal intensity stemming from flow of unsaturated blood into saturated area. The acquired signal intensity is correlated to the degree of blood, hence, TOF MRA technique is mostly used to demonstrate blood flow qualitatively. To quantify the flow reduction, the TOF signal intensity was standardized by division by a reference flow. The minimal standardized flow was recorded as an MRA score to measure and compare occlusion for both the right and left blood vessels.

### Statistical methods

Data are summarized for all animals by mean values. Comparisons were performed using non-parametric statistical tests. Comparisons across groups were performed using the Kruskal-Wallis test and comparisons for paired data were performed using the Wilcoxon signed rank test. Pearson correlations were used to describe relationships between numerical variables.

## Results

### Clinical characteristics of the rats

Sixty rats were included in the study. Four rats died during exposure as follows: one rat following exposure to 609 m for 2 h and 3 rats following exposure to 7620 m for 12 h. One rat died immediately after exposure to 7620 m for 2 h and one control group rat died just before MRI. There was a consistent decrease in weight immediately following exposure to hypobaric conditions, with significant differences by group (*p* < 0.00001, Kruskal-Wallis test). The largest reduction, approximately 20 g on average, occurred in rats that were exposed for 12 h to hypobaric conditions. Weight returned to pre-exposure levels when measured 13 days later.

### Blood counts

**Fig. 1:**
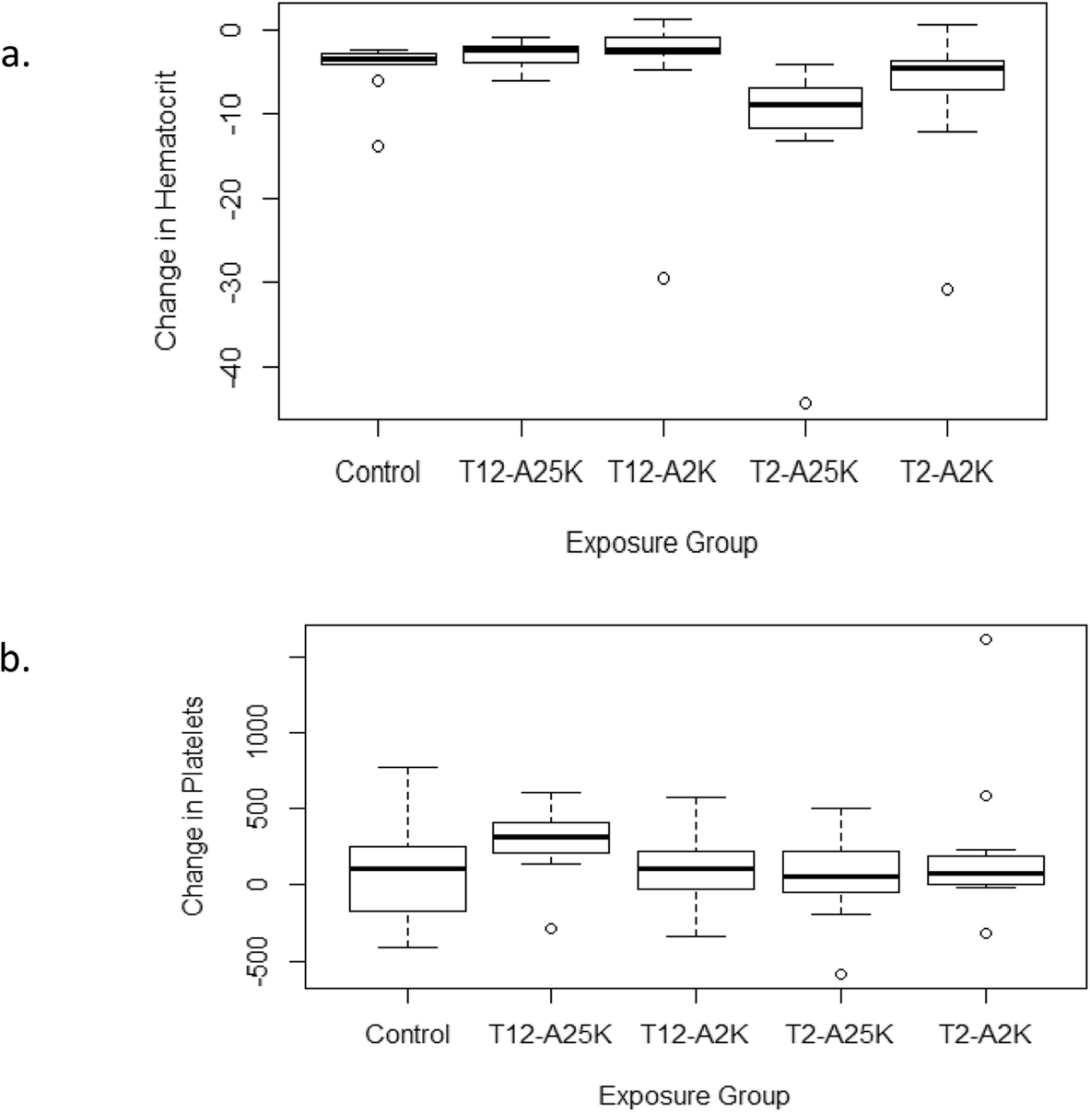
The level of hematocrit (**a**) and platelets (**b**) during acclimation, following exposure to hypobaric conditions and 13 days later according to various hypobaric conditions. T = exposure’s time, A = altitude

There were reductions in both hematocrit and hemoglobin. The reduction in hematocrit differed significantly among the groups (*p* = 0.00016, Kruskal-Wallis test), with the largest reduction observed in rats exposed to 609 m and 7620 m for 2 h as shown in Fig. 1a. There was a weak negative correlation between the change in weight and the change in hematocrit (*r* = − 0.27). The number of platelets increased in 69% of the rats following exposure, with an average increase of 138.3 k/μL. There were no significant differences among the groups (*p* = 0.32, Kruskal-Wallis test) as seen in Fig. 1b.

### TAT and D-dimer

The analysis of TAT complex revealed that five rats were positive at the time of acclimation before exposure to hypobaric conditions (mean ± SD for positive rats: 3020 ± 1692 pg/mL). Following exposure, TAT complex levels were elevated in 8 rats, 4 of which belonged to the control group. No differences in the level of TAT complex were observed between rats exposed to hypobaric conditions and the control group rats (mean ± SD for positive rats: 4900 ± 2125 pg/mL and 4875 ± 3147 pg/mL for control and hypobaric exposure groups, respectively). At 16 days, TAT complexes were elevated in 7 rats: two were exposed to 609 m for 2 h (mean ± SD: 6100 ± 2187 pg/mL), 4 were exposed to 609 m for 12 h (mean ± SD: 5625 ± 5648 pg/mL) and 1 rat was exposed to 7620 m for 2 h (3000 pg/mL). Dot plots of TAT levels at various exposure conditions are presented in supplemental Fig. 1.

D-dimer was negative in all rats during acclimation. Following hypobaric exposure slightly elevated D-dimer levels were observed at 16 days in only two rats (4 ng/ml [609 m for 2 h]; 8 ng/ml [609 m for 12 h]), both of which were negative for TAT complex at all 3 measurements.

### Cytokines: IL-1 and IL-6 levels

The decrease in weight and hematocrit led us to measure IL-1 and IL-6, as both cytokines are known to be involved in inflammation and thrombosis. In 6 rats, the IL-1 levels were already high at the time of acclimation (mean ± SD: 70 ± 46.5 pg/mL). Following exposure, IL-1 was 40 pg/mL in one rat and 80 pg/mL in another one. At the end of the experiment, IL-1 increased in only 2 rats, with levels of 40 pg/mL each.

Elevated IL-6 was found in 4 rats during acclimation (mean ± SD: 238 ± 180 pg/mL). After exposure, IL-6 increased in 3 rats exposed to 609 m for 2 h (mean ± SD: 154 ± 87 pg/mL), in one rat exposed for 12 h (500 pg/mL) and in 2 control rats (300 and 400 pg/mL). At 16 days, IL-6 was elevated in 5 rats, 2 of which were control rats (range 150–400 pg/mL).

Taken together, the increment of IL-1 and IL-6 was not related to mode of exposure or time post-exposure.

### MRA

**Fig. 2:**
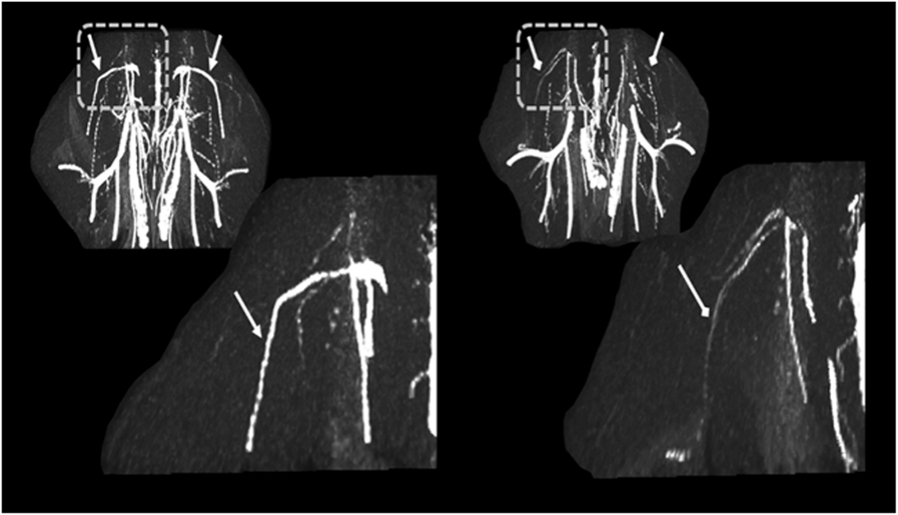
2D-TOF MRI angiography images of the rat’s abdomen area in the coronal view. Two representative rats (N1, N4) with intact and minimal blood flow are given (left and right respectively), with enlargement focusing on the left femoral veins (white arrows). Note the reduction of blood flow in bilateral femoral veins bilaterally, but with different severity

MRA was performed in 54 rats, but could be evaluated in only 52 rats. Figure 2 presents an MRA taken from a representative rat with minimal blood flow in the femoral veins (white arrows) alongside a healthy rat with intact blood flow.

**Fig. 3:**
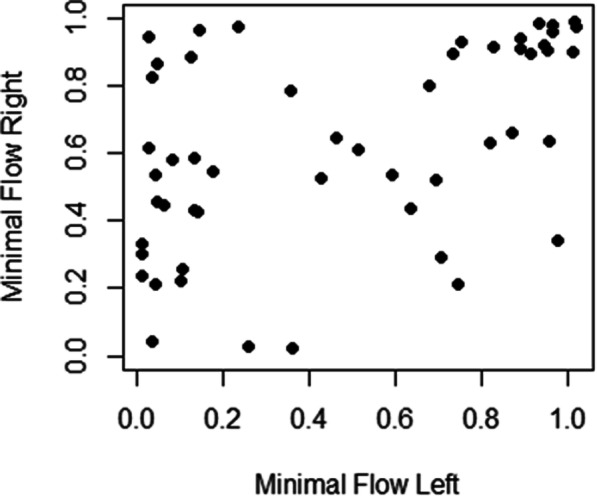
Plot of minimum blood flow (as a fraction of reference flow) in the right vs. the left femur. The line is y = x. Most points are above the line indicating more obstruction in the left vessel. There is a modest positive correlation between the two femurs (*r* = 0.48). Animals with strong reduction in blood flow in the left femur often have little or no reduction in the right femur

Note that the flow deficiency was observed in both right and left femoral veins, but with different severity. Figure 3 shows the modest correlation between minimal flow in the right versus left femoral vein (*r* = 0.48).

Greater flow reduction was observed in the left vessel in 65% of the rats. The median difference of the right vs left flow reduction scores was 0.11 and the differences were statistically different from 0 (*p* = 0.006, Wilcoxon signed rank test).

There were significant differences by exposure group for reduced flow in the right vessel, but not in the left vessel (*p* = 0.002, *p* = 0.150, respectively, Kruskal-Wallis test). The extent of flow reduction did not show any correlation with the exposure conditions. For the right vessel, the lowest median values were 0.36 (for the group exposed for 2 h at 609 m) and 0.46 (12 h at 7620 m). The median values for the other groups were 0.75 (12 h at 609 m), 0.87 (2 h at 7620 m) and 0.90 (control). The left vessel had larger flow reductions; the lowest median values were 0.13 (12 h at 7620 m), 0.14 (2 h at 609 m) and 0.28 (control). The median values for the other groups were 0.69 (2 h at 7620 m) and 0.82 (12 h at 609 m).

Half of the rats (26/52) had serious blood flow reduction to 0.3 or less in at least one of the two femoral veins. By group, these fractions ranged from 27% (3/11) in the 2 h, 7620 m group to 80% (8/10) in the 2 h, 609 m group, with 50% (5/10) in the control group.

## Discussion

In this study of a rat model of exposure to different hypobaric conditions and duration, designed to mimic the conditions prevailing during civil flights or military aircraft missions, we found no laboratory indications for the activation of coagulation and fibrinolytic systems. Indeed, the amount of thrombin generated, which is often assessed by TAT complex, and the fibrinolytic system activity, which is reflected by increased D- dimer levels, were not elevated in most animals immediately following exposure or 13 days later.

In fact, when D-dimer was measured in cockpit crews who fly multiple short duration flights to rule out subclinical thrombotic events, there was no evidence for an increase [15]. Others assumed that the failure to detect raised concentration of D-dimer in passengers with positive ultrasound scans is related to the short half-life (about 6 h) of D-dimer and the long delay (up to 48 h) before blood was sampled upon return from travel [16]. Therefore, in our project, we withdrew blood immediately post exposure and after 13 days, taking into account the fact that the risk of DVT tends to increase in the first 2 weeks following the flight [4, 11].

Additionally, we found reductions in hemoglobin and hematocrit, despite the fact that the rats were water deprived. Similar observations were noticed in 20 volunteers after transatlantic flight, where hemoglobin decreased without signs of dehydration [7].

A significant weight loss was observed in rats immediately post-exposure, with weight regained when analyzed 13 days later. The loss of weight following hypobaric exposure is attributed to muscle atrophy caused by increased protein degradation rate through up regulation of the ubiquitin-proteasome pathway [17]. This mechanism cannot explain the regained weight we observed in our rat model at day 13-post exposure or the decrease in weight in the control group. This fact led us to assess IL-1 and IL-6, both inflammatory cytokines that may be involved in thrombosis, besides being involved in the regulation of body fat [18, 19].

The level of IL-1 and IL-6 increased post-exposure in a small number of rats, but it was not related to exposure conditions.

We speculated that by using a 7 T/30 MRA, we might reach higher diagnostic accuracy for detecting vessel occlusion in rats at 13 days post exposure. A significant reduction of blood flow to less than 30% in 26 rats (50%) was observed in the femoral vein. These reductions were not related to exposure conditions.

Furthermore, we compared the blood flow between the right and left side of the lower limbs in all the rats and in relation to their group to rule out some slowdown of the flow that may indicate the likelihood of imminent clot formation. Occlusion of the left femoral vein was significantly greater than that of the right. Occlusion only of the right vessel achieved statistically significant differences across groups, but the extent of occlusion in that vessel was again unrelated to the exposure conditions.

We realize that our rat model cannot replace a human being, but we found that the aircraft cabin environment with hypobaric conditions did not promote vessel occlusion. This may explain why the incidence of DVT in pilots is not increased, even if they fly more frequently than most passengers [20].

We are aware of the fact that we did not have real-time measurements of partial oxygen pressure during exposure to of 609 m and 7620 m, but used estimated pressures corresponding to the altitudes, reaching 19.4 and 7.6% respectively.

Since the coagulation and fibrinolysis pathways were not found to be activated, the degree of hypoxia should not alter our results.

## Conclusion

In summary, our rat model points out that in hypobaric conditions simulating civil and combat flights to a certain degree, there was no evidence of substantial activation of coagulation, fibrinolysis, increased inflammatory cytokines or alteration of blood flow correlated with the type of exposure.

## Supplementary information

**Additional file 1: Supplementary Fig. 1**. TAT levels in rats allocated to the control group (sea level, no hypobaric exposure) and various hypobaric exposure groups (609 m 2 h, 609 m 12 h, 7620 m 2 h, 7620 m 12 h). For selected individual rats, changes in TAT levels from baseline are illustrated by lines of different colors, with each color representing an individual rat examined at various time points: baseline, immediately post-exposure and 16 days post-exposure.

## Data Availability

All data generated or analysed during this study are included in this published article (and its supplementary information files).

## Abbreviations

TAT: thrombin-antithrombin
DVT: deep vein thrombosis
ELISA: enzyme-linked immunosorbent assay
2D-TOF: 2-dimensional Time of Flight
MRA: MRI angiography
