# Supplementary figures and images for: The effect of civil and military flights on coagulation, fibrinolysis and blood flow: insight from a rat model
Source: Thromb J. 2020 Oct 6;18:24. doi: 10.1186/s12959-020-00237-8 (PMC7541254; doi:10.1186/s12959-020-00237-8)

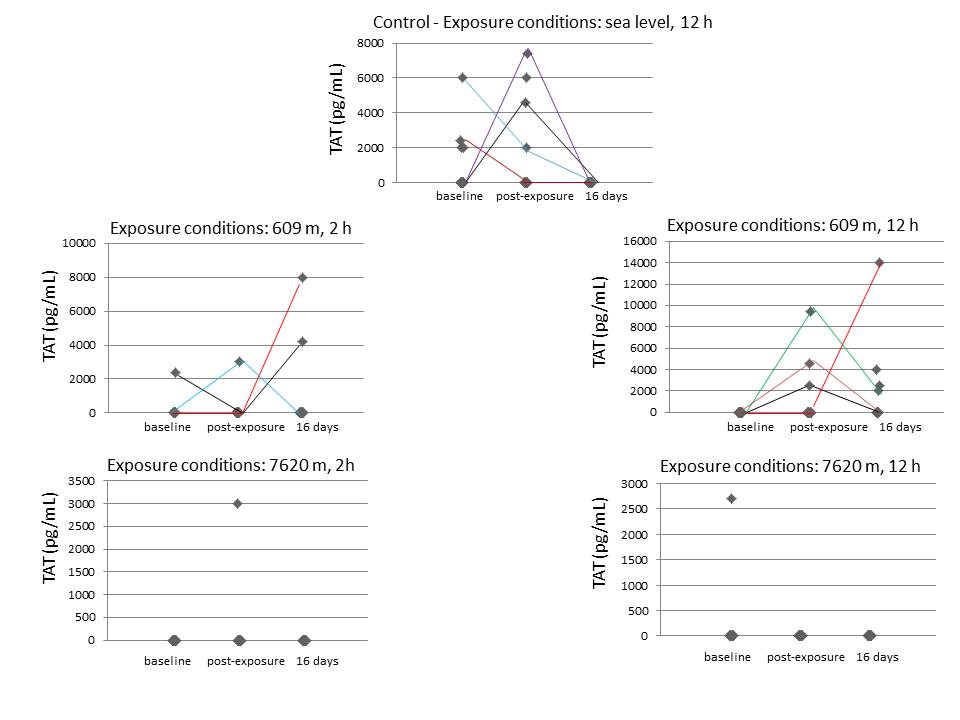

Supplement: Supplementary file 1 — Additional file 1: Supplementary Fig. 1. TAT levels in rats allocated to the control group (sea level, no hypobaric exposure) and various hypobaric exposure groups (609 m 2 h, 609 m 12 h, 7620 m 2 h, 7620 m 12 h). For selected individual rats, changes in TAT levels from baseline are illustrated by lines of different colors, with each color representing an individual rat examined at various time points: baseline, immediately post-exposure and 16 days post-exposure. [file 12959_2020_237_MOESM1_ESM.jpg]
